# Antibacterial efficacy of ultrahigh molecular weight polyethylene with silver containing diamond-like surface layers

**DOI:** 10.1186/s13568-015-0148-x

**Published:** 2015-09-21

**Authors:** Norbert Harrasser, Sebastian Jüssen, Ingo J. Banke, Ralf Kmeth, Ruediger von Eisenhart-Rothe, Bernd Stritzker, Hans Gollwitzer, Rainer Burgkart

**Affiliations:** Clinic of Orthopedics and Sports Orthopedics, Klinikum rechts der Isar, Technical University of Munich, Ismaninger Str. 22, 81675 Munich, Germany; Experimental Physics IV, Institut für Physik, Augsburg University, Universitätsstr. 1, 86135 Augsburg, Germany; ATOS Clinic, Effnerstr. 38, 81925 Munich, Germany

**Keywords:** Implant-associated infections, Diamond-like carbon, Silver, *Staphylococcus epidermidis*, Antibacterial coating

## Abstract

Antibacterial coating of medical devices is a promising approach to reduce the risk of infection but has not yet been achieved on wear surfaces, e.g. polyethylene (PE). We quantitatively determined the antimicrobial potency of different PE surfaces, which had been conversed to diamond-like carbon (DLC-PE) and doped with silver ions (Ag-DLC-PE). Bacterial adhesion and planktonic growth of various strains of *S. epidermidis* on Ag-DLC-PE were compared to untreated PE by quantification of colony forming units on the adherent surface and in the growth medium as well as semiquantitatively by determining the grade of biofilm formation by scanning electron microscopy. (1) A significant (p < 0.05) antimicrobial effect could be found for Ag-DLC-PE. (2) The antimicrobial effect was positively correlated with the applied fluences of Ag (fivefold reduced bacterial surface growth and fourfold reduced bacterial concentration in the surrounding medium with fluences of 1 × 10^17^ vs. 1 × 10^16^ cm^−2^ under implantation energy of 10 keV). (3) A low depth of Ag penetration using low ion energies (10 or 20 vs. 100 keV) led to evident antimicrobial effects (fourfold reduced bacterial surface growth and twofold reduced bacterial concentration in the surrounding medium with 10 or 20 keV and 1 × 10^17^ cm^−2^ vs. no reduction of growth with 100 keV and 1 × 10^17^ cm^−2^). (4) Biofilm formation was decreased by Ag-DLC-PE surfaces. The results obtained in this study suggest that PE-surfaces can be equipped with antibacterial effects and may provide a promising platform to finally add antibacterial coatings on wear surfaces of joint prostheses.

## Introduction

The great success of surgically-implanted biomaterials may be compromised in every case by the challenging complication of bacterial periimplant infection (Liu et al. 2012; Zimmerli and Ochsner 2003). Approximately 2.6 million orthopedic biomaterials are implanted annually in the USA, hence the incidence of implant-associated infections is also increasing (Kurtz et al. 2008). Most important in the pathogenesis of infection is the colonization of the device surface and the consecutive formation of a biofilm (Zimmerli and Moser 2012; Gosheger et al. 2004), in which *Staphylococcus aureus* and *koagulase*-*negativeStaphylococci* are most frequently implicated as the etiologic agents (Zimmerli and Ochsner 2003; Hunter and Dandy 1977). Prevention of these infections has an important impact not only on patient’s morbidity but also on the cost effectiveness of hospital care (Gosheger et al. 2004). Systemic antibiotic prophylaxis and various local antibiotic delivery techniques have been proven to reduce the rate of infection (Gollwitzer et al. 2003; Schmidmaier et al. 2006). Hereby locally applied antibiotics are advantageous in delivering high drug concentrations to the required site without producing systemic toxicity (Zhang et al. 2014). Because pathogens involved in implant associated infections are diverse and bacteria in biofilms are protected from antibiotics (Ceri et al. 1999), the restricted activity of these substances limits their clinical effectiveness, especially in infections involving antibiotic-resistant bacterial strains (e.g. MRSA) (Liu et al. 2012). Therefore, alternatives to local antibiotic delivery systems are highly favored. In this context employment of implant materials or coatings that control infection and biofilm formation would be particularly advantageous (Schmidmaier et al. 2006). This led to the development of antiadhesive and non-antibiotic antibacterial surfaces. The first mentioned coatings (e.g. polyethylene glycol, polyethylene oxide brushes) reduce bacterial adhesion by altering the physicochemical properties of the substrate. Thus, formation of protein surface layers (conditioning films) on the implant and bacteria–substrate interactions are hindered (Hetrick and Schoenfisch 2006). This mode of action is referred to as ‘‘passive’’. However the effectiveness of these coatings for reducing bacterial adhesion is very limited and varies greatly depending on bacterial species. Additionally, osseointegration is poor. In sum, the importance of these antiadhesive coatings in orthopedic surgery is limited. In contrast non-antibiotic “active” antibacterial coatings release antibacterial agents, e.g. silver ions (Ag^+^), copper ions (Cu^++^), nitric oxide, chlorhexidine/chloroxylenol or chitosan (Kumar and Munstedt 2005; Hardes et al. 2007; Gosheger et al. 2004; Shirai et al. 2009). Compared to antibiotics these agents act more broadly against a wide range of bacteria. In addition, at least proven for the use of Ag^+^, microbes without intrinsic resistance cannot gain resistance (Kumar and Munstedt 2005; Lee et al. 2005).

So far, these antibacterial coatings have not been applied on soft wear surfaces, e.g. polyethylene (PE). In total knee replacement roughly half of the surface is exposed to synovial fluid and in main parts tribologically active. Therefore in septic revision surgery major portions of the susceptible prosthesis are not protected against bacterial reinfection. Antibacterial-agent-enriched diamond-like carbon (DLC) surfaces may solve this dilemma. By release of Ag^+^ these surfaces could act as local antibacterial agents (Cloutier et al. 2014; Katsikogianni et al. 2006). At the same time appropriate DLC surfaces can exhibit excellent tribological features as already shown for hip or knee arthroplasty (Saikko et al. 2001; Dearnaley 1993; Oliveira et al. 2014).

In this study the antimicrobial effects of silver (Ag) incorporated DLC surfaces on PE (Ag-DLC-PE) are investigated. PE was chosen due to its outstanding importance in orthopedic surgery as a wear surface. This study provides valuable information for determining the suitability of Ag-DLC-PE for septic revision surgery.

## Materials and methods

### Study substrates and surface conversion

**Fig. 1:**
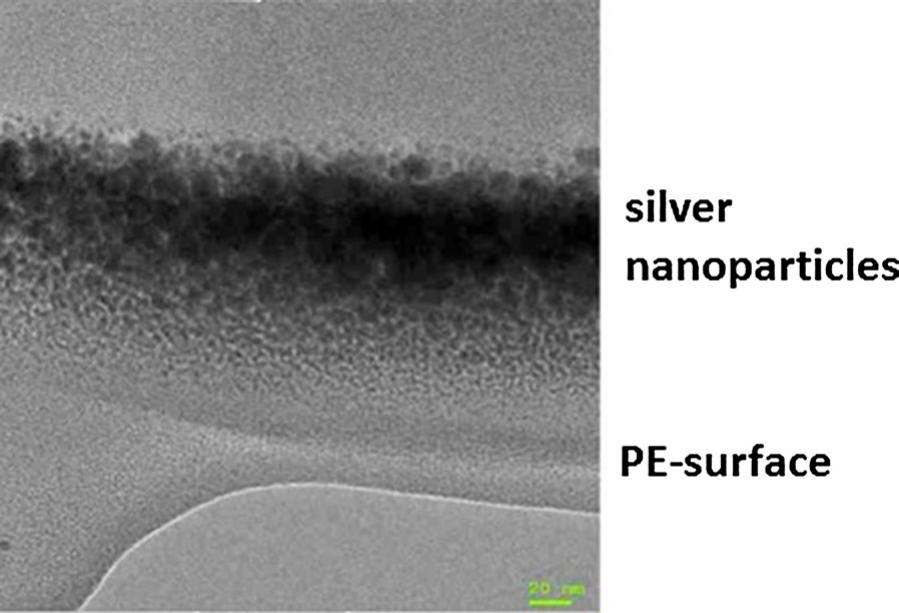
Transmission electron microscopy (TEM) image of silver nanoparticles of AG-DLC-PE; *notice* nanoparticles do not coat the PE surface but are embedded in the PE

Study objects were cylindrical substrates (diameter: 10 mm, height: 2 mm; Goodfellow GmbH, Nauheim, Germany) of PE (ultrahigh molecular weight PE, UHMWPE). DLC-processing of the plates was performed at the Department of Experimental Physics IV, University of Augsburg (Germany). The samples were treated according to a modified technique of ion irradiation of polymers, in which DLC-processing was achieved by direct ion bombardment of Ag^+^ or nitrogen (Bertóti et al. 2007). In contrast to common DLC techniques, the PE surface is not coated with DLC but rather modified by silver ion implantation. Due to the kinetic energy of the implanted Ag^+^, the polymer surface is modified from crystalline PE to amorphous DLC, while the metal ions agglomerate to Ag nano-particles directly under the surface. In this way, the implantation of silver ions leads to a wear-resistant, silver-containing modified PE surface reducing the risk of detachment compared to surface coatings (Fig. 1) (Schwarz and Stritzker 2010).

These surface conversed DLC-PE samples were investigated in three groups with modified parameters of Ag^+^-implantation: firstly, to determine the influence of different ion energies (first group) and secondly, to determine the influence of different fluences (second group). In order to elucidate which of these factors (ion energy vs. fluence) has a major impact on microbes a third testing group was conducted. All sample features and testing groups are given in Table 1.

**Table 1:** Physical parameters of DLC conversion and antibacterial effect of different Ag-DLC-PE surfaces compared to untreated PE

Implantation energy, fluence	Surface adhesion (CFU; mean ± SD)	Bacterial growth of Ag-DLC-PE (log-levels^a^/%)^b^	p values	Planktonic growth (CFU/ml; mean ± SD)	Bacterial growth of Ag-DLC-PE (log-levels^a^/%)^b^	p values
Constant fluences (1st testing group)						
100 keV. 1 × 10^17^ cm^−2^	3.2 × 10^4^ ± 3.3 × 10^4^	+0.25/+77.8 %	0.901	4.5 × 10^5^ ± 3.0 × 10^5^	+0.5/+200 %	<0.05
80 keV. 1 × 10^17^ cm^−2^	1.7 × 10^4^ ± 1.4 × 10^4^	−0.02/−5.6 %	0.252	2.8 × 10^5^ ± 1.3 × 10^5^	+0.3/+86.7 %	<0.05
60 keV. 1 × 10^17^ cm^−2^	2.6 × 10^3^ ± 2.5 × 10^3^	−0.8/−85.6 %	<0.05	1.7 × 10^5^ ± 8.5 × 10^4^	+0.05/+13.3 %	0.884
Untreated PE	1.8 × 10^4^ ± 9.4 × 10^3^			1.5 × 10^5^ ± 2.8 × 10^4^		
Constant implantation energies (2nd testing group)						
10 keV. 1 × 10^16^ cm^−2^	2.7 × 10^4^ ± 9.8 × 10^3^	−0.03/−6.9 %	0.821	6.1 × 10^5^ ± 2.0 × 10^5^	+0.3/+103.3 %	<0.05
10 keV. 5 × 10^16^ cm^−2^	6.8 × 10^3^ ± 6.2 × 10^3^	−0.6/−76.6 %	<0.05	1.4 × 10^5^ ± 1.1 × 10^5^	−0.3/−53.3 %	<0.05
10 keV. 1 × 10^17^ cm^−2^	9.6 × 10^2^ ± 8.5 × 10^2^	−1.5/−96.7 %	<0.05	1.5 × 10^4^ ± 1.2 × 10^4^	−1.3/−95 %	<0.05
Untreated PE	2.9 × 10^4^ ± 2.0 × 10^4^			3.0 × 10^5^ ± 6.5 × 10^4^		
Fluence vs. implantation energies (3rd testing group)						
20 keV. 5 × 10^16^ cm^−2^	4.0 × 10^3^ ± 3.5 × 10^3^	−1.0/−89.2 %	<0.05	5.8 × 10^4^ ± 1.9 × 10^4^	−0.4/−55.4 %	<0.05
20 keV. 1 × 10^17^ cm^−2^	2.0 × 10^3^ ± 1.6 × 10^3^	−1.3/−94.6 %	<0.05	3.0 × 10^4^ ± 1.3 × 10^4^	−0.6/−76.9 %	<0.05
10 keV. 5 × 10^16^ cm^−2^	3.9 × 10^3^ ± 2.8 × 10^3^	−1.0/−89.6 %	<0.05	6.4 × 10^4^ ± 4.8 × 10^4^	−0.3/−50.8 %	<0.05
10 keV. 1 × 10^17^ cm^−2^	2.3 × 10^3^ ± 1.9 × 10^3^	−1.2/−93.8 %	<0.05	3.6 × 10^4^ ± 2.3 × 10^4^	−0.6/−72.3 %	<0.05
Untreated PE	3.7 × 10^4^ ± 2.8 × 10^4^			1.3 × 10^5^ ± 3.4 × 10^4^		

*fluence* amount of ions received by a surface per unit area (ions/cm^2^), *CFU* colony forming units, *SD* standard deviation

^a^log-levels = bacterial counts calculated as shown in following equation: log-levels = log_10_(CFU of Ag-DLC-PE) − log_10_(CFU of untreated PE)

^b^Positive values (log-levels/%) express increased bacterial growth on Ag-DLC-PE compared to PE, negative values express reduced bacterial growth on Ag-DLC-PE compared to PE

In the first group three Ag-doped samples with constant fluences (1 × 10^17^ cm^−2^) and different ion energies (60, 80, 100 keV) were assembled. DLC processing was carried out via direct ion implantation of Ag^+^. Based on the findings of the first group the second group was performed with three different fluences of Ag^+^ (1 × 10^16^, 5 × 10^16^ and 1 × 10^17^ cm^−2^) and constant low implantation energy (10 keV). In the third group samples were subjected to ion bombardment of Ag^+^ with different fluences and low ion energies (20 keV: 5 × 10^16^ and 1 × 10^17^ cm^−2^; 10 keV: 5 × 10^16^ and 1 × 10^17^ cm^−2^). Non-modified PE samples served as a control.

After sample preparation incubation for 24 h with *Staphylococcus epidermidis* (ATCC35984) was carried out. Thereafter, antimicrobial effects on the sample’s surface (i.e. bacterial sessile growth) and the surrounding fluid medium (i.e. bacterial planktonic growth) were investigated.

### Sterilization of samples and sealing of surfaces with paraffin wax

**Fig. 2:**
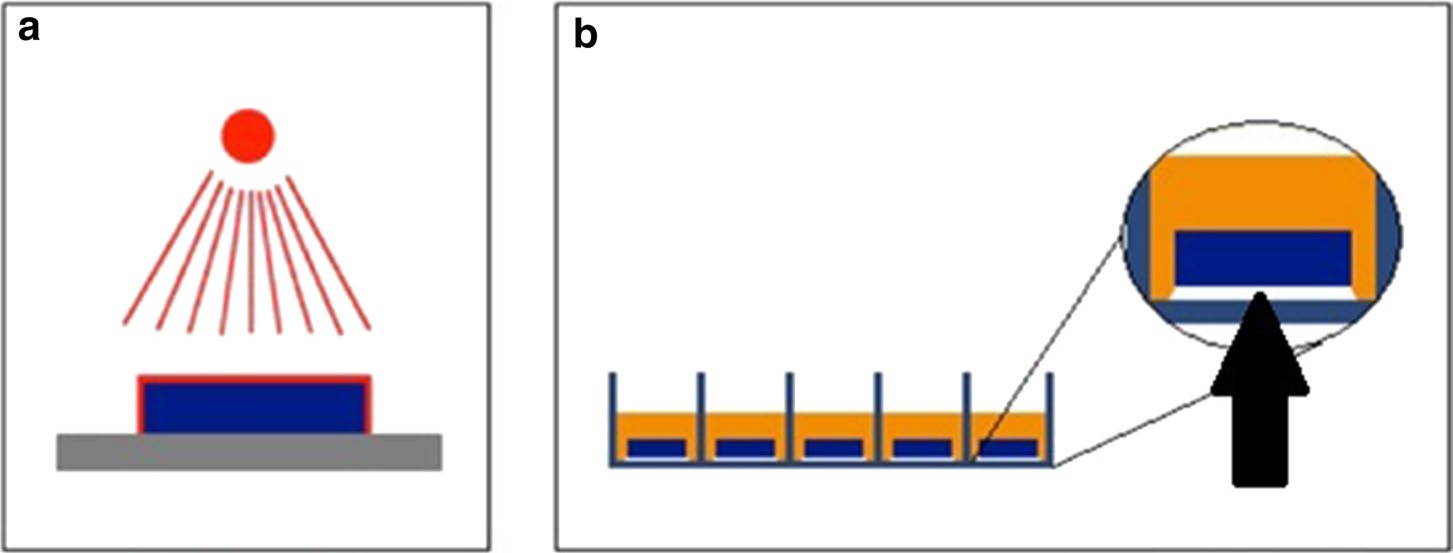
Sample preparation; ion irradiation of samples with missing irradiation of the sample’s lower surfaces (**a**), placement of samples in well culture plates with paraffin wax (*arrow*) covering the sample’s lower surface (**b**)

Samples were rinsed with distilled water for 10 min, air-dried in a laminar flow cabinet and thereafter sterilized with gamma-beam with the dose of 26.5 kGy (Isotron Deutschland GmbH, Allershausen, Germany). All manipulations of the samples were conducted by holding the lower surface. As a consequence these parts of the samples were not surface treated and needed protection from the testing environment. Hence, paraffin wax was first autoclaved in a glass container with 120 °C for 20 min (Varioklav^®^, H + P Labortechnik, H + P Labortechnik AG, Oberschleißheim, Germany), the samples’ lower surfaces were then dip-coated in the solvent paraffin wax so that a thin protection layer was formed. Specimens were then placed in 24-well culture plates (Fig. 2a, b). Pretesting with paraffin wax revealed no intrinsic antimicrobial potential and was therefore appropriate as a mechanical sample stabilizer.

### Bacterial sample preparation

The bacterial strains used in the present study were *S. epidermidis* (ATCC 35984; LGC Standards GmbH, Wesel, Germany) for determination of surface and planktonic growth and a strong biofilm-forming variant of *S. epidermidis* (RP62a; LGC Standards GmbH, Wesel, Germany) for scanning electron microscopy (SEM-) evaluation of biofilm formation on the samples. These strains are known for their outstanding significance in implant-associated infections (Zimmerli and Ochsner 2003; Darouiche 2004). Test strains were routinely cultured in Columbia Agar with 5 % sheep blood (*S. epidermidis*, ATCC 35984) or Trypticase™ Soy Agar (*S. epidermidis*, RP62a) (Becton–Dickinson, Heidelberg, Germany) at 37 °C overnight before testing. Bacteria were then harvested by centrifugation, rinsed, suspended, diluted in sterile phosphate buffered saline (PBS) and adjusted by densitometry to a MacFarland 0.5 standard (MacFarland Densimat™, BioMérieux, Marcy l’Etoile, France). To control bacterial concentration, 100 μl of each suspension was again cultured for 24 h at 37 °C. After 24 h serial dilutions of this suspension were plated on Colombia-Agar. The colonies were counted and colony numbers calculated accordingly. For the study every suspension with its known bacterial concentration was diluted with DMEM + 10 % FCS to reach the targeted value for bacterial concentration (10^5^ CFU/ml). Sample plates with paraffin-coated lower surfaces were placed in 24-well culture plates and 1 ml of 10^5^ CFU/ml bacterial suspensions were added. Incubation of the well plates was conducted for 24 h at 37 °C.

### Analysis

Bacterial surface adhesion was evaluated by determining bacterial concentration on the specimen. Bacterial planktonic growth was measured in the growth medium. For every group four independent testing runs with four different samples were conducted. Therefore, altogether 16 samples were tested for every group.

### Determination of bacterial growth on sample surfaces

Colonized sample plates were removed from the wells with a sterile forceps, carefully rinsed twice with sterile PBS, transferred to vials containing 3 ml of sterile PBS and sonicated for 7 min (Elmasonic S60H, Elma, Singen, Germany) to remove adhering bacteria. 100 μl of the fluid were aspirated, plated on Colombia Agar at 37 °C for 24 h and quantified after incubation (CFU/ml).

Scanning electron microscopy-analysis was conducted semiquantitatively to evaluate inhibition of biofilm formation. SEM-images were compiled of native DLC coated PE samples and Ag-DLC-PE samples. Biofilm formation was quantified in five categories: (1) no biofilm formation, (2) biofilm covering less than 25 % of the surface, (3) biofilm covering between 25 and 75 % of the surface, (4) biofilm covering more than 75 % of the surface, (5) biofilm formation covering the entire surface.

### Determination of bacterial planktonic growth

A 700-μl volume of each well was supplemented with 700 μl neutralizing solution as described by Tilton and Rosenberg (1978) (1.0 g sodium thioglycolate + 1.46 g sodium thiosulfate in 1.000 ml deionized water). The neutralizing solution acts as an inhibitor for reminiscent metal toxicity on bacteria. The suspension was plated on Columbia Agar after serial dilutions and incubated at 37 °C for 24 h. Thereafter, CFU were quantified and extrapolated to CFU/ml.

### Statistics

All results are presented as mean ± standard deviation. Statistical significance was computed using non-parametric methods and the method of closed testing procedure (Kruskal–Wallis and Mann–Whitney U test). P < 0.05 was considered statistically significant. Statistical tests were performed with use of SPSS (version 20.0; Chicago, IL, USA). Statistical analysis was conducted per consultation with the Institute of Medical Statistics and Epidemiology (Klinikum rechts der Isar, Technische Universität München, Munich, Germany).

## Results

### Antimicrobial effect of Ag-DLC-PE with different high ion energies (60, 80, 100 keV) and equal fluences (1 × 10^17^ cm^−2^): testing group 1

**Fig. 3:**
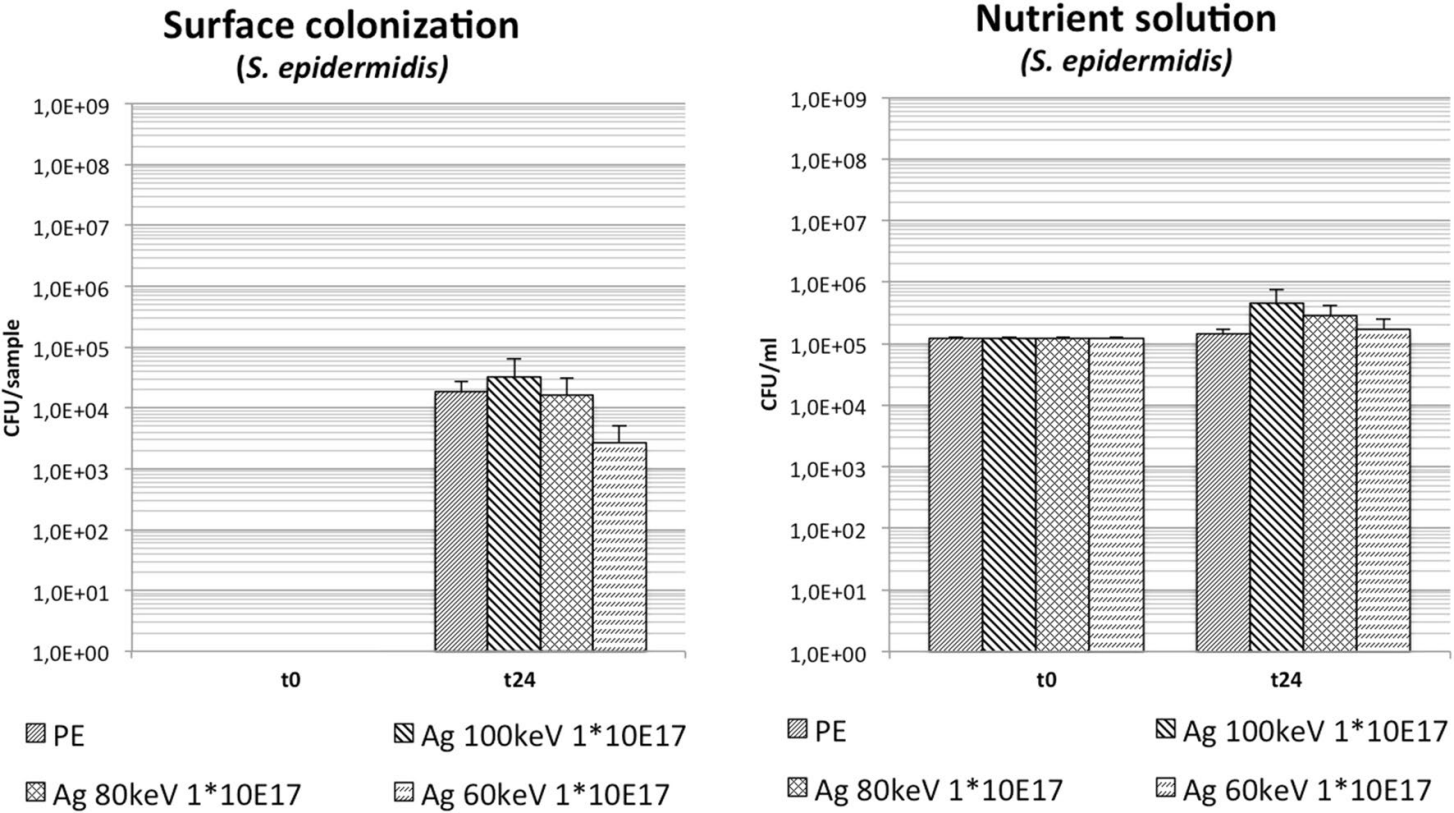
Bacterial growth of *S. epidermidis* in the Ag-DLC-PE testing group 1 with constant fluences and different implantation energies (t = 0: before incubation; t = 24 h: after incubation)

Compared to non-treated PE samples a minimally increased bacterial surface adhesion was found on samples after DLC conversion and Ag^+^ implantation with 100 keV. A comparable finding was observed with samples treated with 80 keV. However, on Ag-DLC-PE samples treated with only 60 keV a significantly and clinically relevant decreased bacterial growth was evident (Table 1; Fig. 3). Analysis of planktonic growth in the supernatant growth medium showed significantly increased bacterial concentrations for samples treated with 100 and 80 keV compared to PE; samples conversed with 60 keV did not show a significant increase of bacterial growth (Table 1; Fig. 3).

### Antimicrobial effect of Ag-DLC–PE with different fluences (1 × 10^16^, 5 × 10^16^ and 1 × 10^17^ cm^−2^) and constant low ion energy (10 keV): testing group 2

**Fig. 4:**
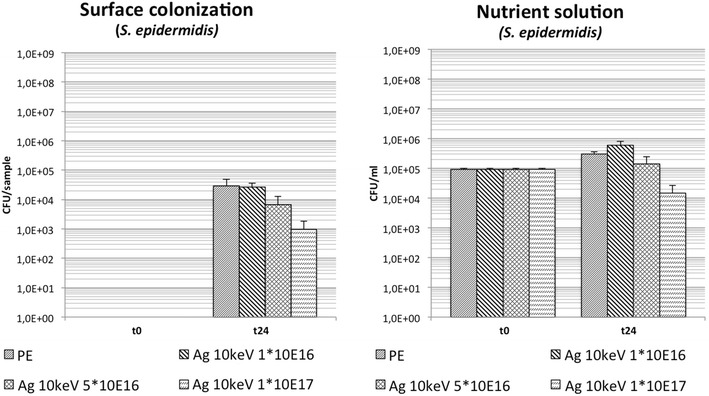
Bacterial growth of *S. epidermidis* in the Ag-DLC-PE testing group 2 with constant low implantation energies and different fluences (t = 0: before incubation; t = 24 h: after incubation)

Ag-DLC-PE showed a decreased bacterial surface adhesion compared to PE by 0.03 log-levels (p > 0.05) for fluences of 1 × 10^16^ cm^2^, by 0.6 log-levels (p < 0.05) with fluences of 5 × 10^16^ cm^−2^ and by 1.5 log-levels (p < 0.05) on samples with the highest fluences (1 × 10^17^ cm^−2^). Analysis of planktonic growth showed minimally increased bacterial concentrations compared to untreated PE only in broths containing samples with fluences of 1 × 10^16^ cm^−2^. Surface conversion with Ag and fluences of 5 × 10^16^ and 1 × 10^17^ cm^−2^ resulted in a reduction of planktonic bacterial growth (Table 1; Fig. 4).

### Low ion energy (10, 20 keV) vs. fluence (5 × 10^16^ and 1 × 10^17^ cm^−2^): comparison of these two features regarding the antimicrobial effect of Ag-DLC-PE: testing group 3

**Fig. 5:**
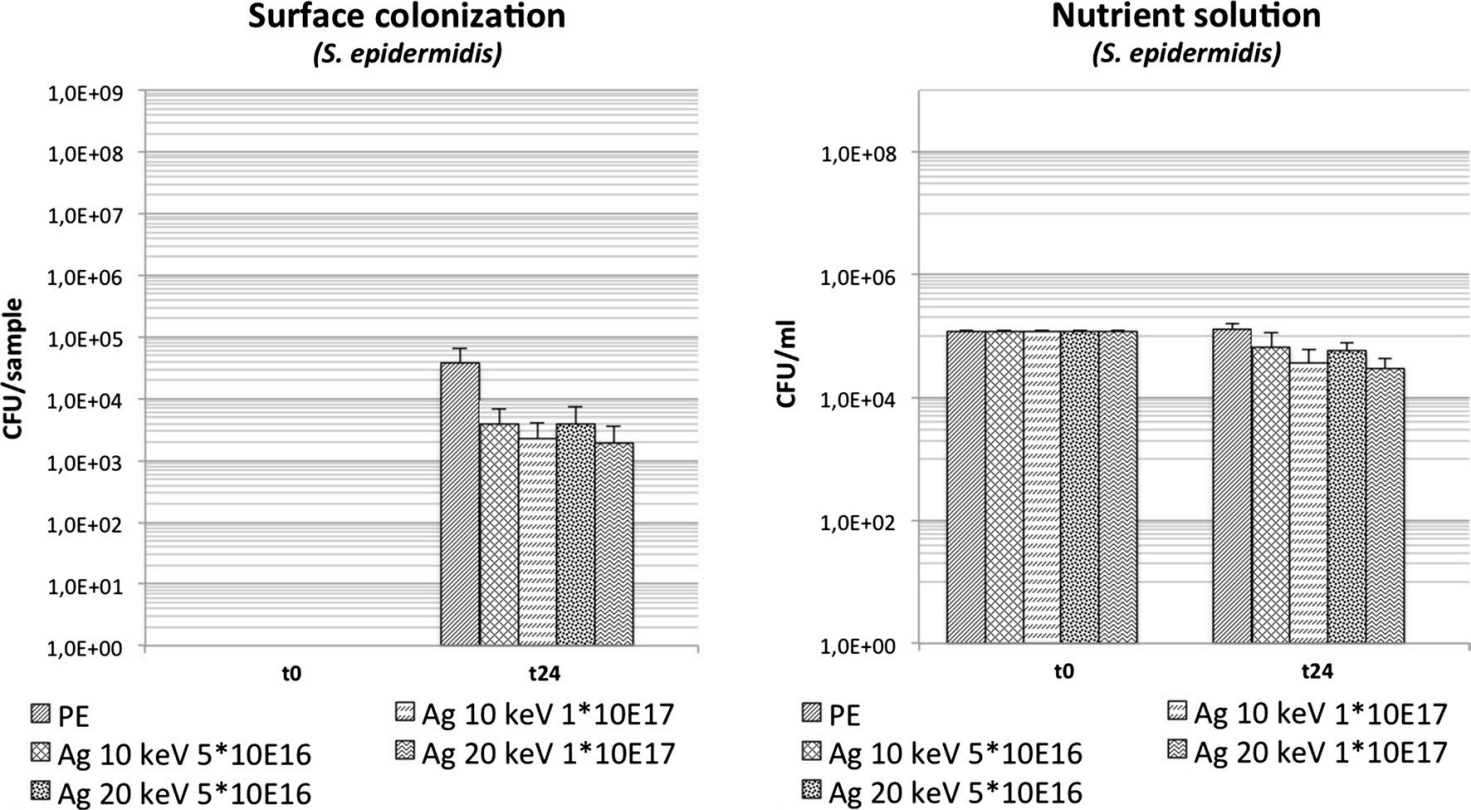
Bacterial growth of *S. epidermidis* in the Ag-DLC-PE testing group 3 with comparison of different fluences vs. different implantation energies (t = 0: before incubation; t = 24 h: after incubation)

Analysis of surface adhesion on Ag-DLC-PE samples conversed with 10 keV ion energy implantation showed a significant reduction of bacterial growth on specimen treated with fluences of 5 × 10^16^ and 1 × 10^17^ cm^−2^. Similarly, samples treated with 20 keV ion energy implantation showed significantly decreased bacterial growth on samples with fluences of 5 × 10^16^ and 1 × 10^17^ cm^−2^ (Table 1; Fig. 5). Analysis of planktonic growth of samples treated with 20 keV ion energy implantation showed significantly decreased bacterial concentrations with fluences of 5 × 10^16^ and 1 × 10^17^ cm^−2^. Samples with 10 keV ion energy implantation and fluences of 5 × 10^16^ and 1 × 10^17^ cm^−2^ also showed a reduction of bacterial growth (Table 1; Fig. 5).

### Surface biofilm formation in scanning electron micrographs

**Fig. 6:**
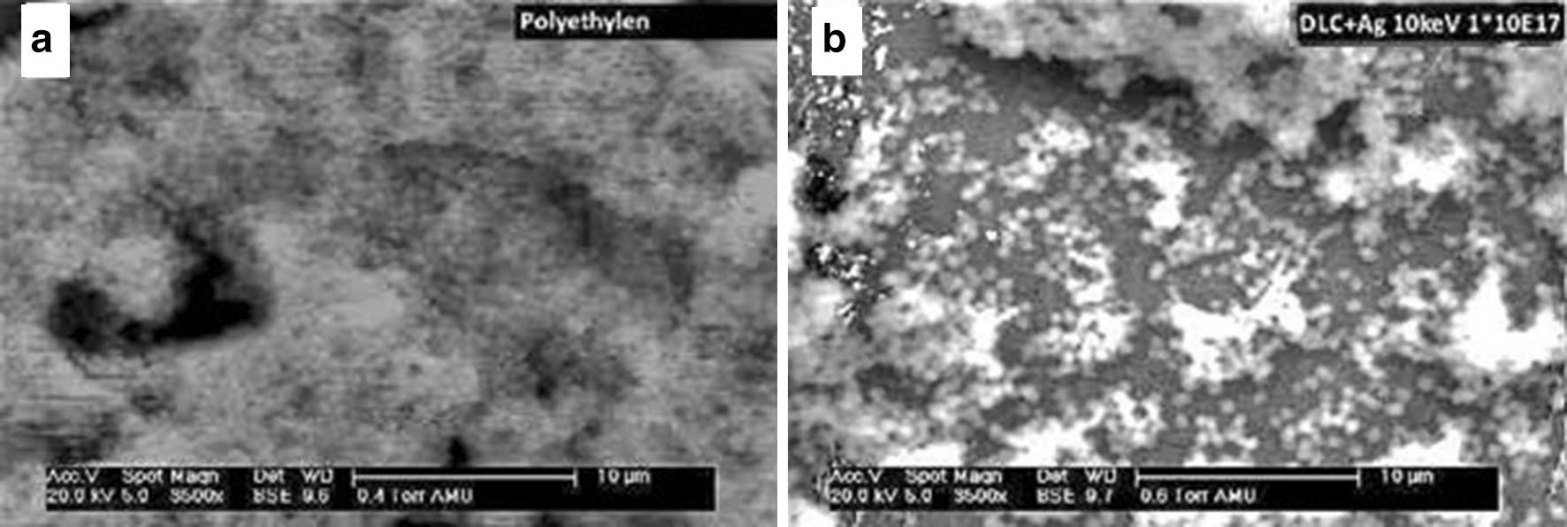
Biofilm formation on different polyethylene surfaces. Homogenous biofilm grade 5 after incubation with *S. epidermidis* on native PE (**a**), reduced biofilm grade 3 on Ag-DLC-PE (**b**)

Biofilm formation was ubiquitous and graded type 5 on all pure PE samples without Ag incorporation covering the entire specimen surfaces with thick layers of *S. epidermidis*. Ag-DLC-PE samples on the other hand showed biofilm inhibiting effects with at the most rare spot-like biofilm formation graded type 3 (Fig. 6a, b).

## Discussion

Recent strategies to lower periimplant infection rates are based on the primary prevention of bacterial adhesion by non-adhesive coatings (Groll et al. 2009; Harris et al. 2004) or impairment of bacterial survival and biofilm formation by surface coatings releasing non-antibiotic organic antimicrobial agents like chlorhexidine or chitosan (Bumgardner et al. 2003; Verraedt et al. 2011; Baffone et al. 2011) and inorganic antimicrobial agents like Ag^+^, Cu^++^ or nitric-oxide (Zhao et al. 2011; Fiedler et al. 2011; Holt et al. 2011; Kumar and Munstedt 2005). To our best knowledge, no attempt has been conducted so far to apply these coatings on soft wear surfaces, e.g. PE. This leads to a major unprotected surface area of joint prostheses favoring reinfection, especially in septic revision surgery. To solve this problem addition of bactericidal agents to DLC surface modifications could be promising, based on the finding that DLC applied at PE is known to exhibit excellent wear behavior (Saikko et al. 2001; Dearnaley 1993; Oliveira et al. 2014). Some studies investigated Ag doped DLC coatings on hard wear surfaces e.g. steel and found significant bactericidal effects (Soininen et al. 2011; Marciano et al. 2009; Katsikogianni et al. 2006; Kwok et al. 2007; Baba et al. 2013). To our knowledge, no report has been published so far describing antibacterial conversion of PE for antibacterial purposes.

Ag seems to be of outstanding value in the prevention and treatment of implant associated infections (Morones et al. 2005; Taglietti et al. 2012; Hardes et al. 2010). Ag acts by binding to membranes, enzymes and nucleic acids. Consequently the respiratory chain is inhibited and therefore the aerobe metabolism of microorganisms disturbed (Gosheger et al. 2004). Bacteria are quite susceptible to Ag with only negligible possibility of intrinsic resistance (Kumar and Munstedt 2005). Antibacterial effects have been reported to be directly proportional to Ag concentrations and therefore directly depend on Ag release into the surrounding environment (Schierholz et al. 1998; Morones et al. 2005). These findings were confirmed in the present study (Table 1). In this context, important properties of the tested coatings could be identified: it was found that antimicrobial efficacy on the surface of Ag-DLC-PE treated with high energies of ion implantation (60–100 keV) was only significant in samples treated with ion energies of 60 keV. No bactericidal effect in this setting was determined in the surrounding medium. From a physical point of view this is not surprising since high ion energies determine a rather deep implantation of Ag preventing the atoms from release into the surrounding medium. This “deep depositioning” effect of DLC surfaces on ions implanted with high energies has already been described in the literature in other materials than PE (Furno et al. 2004). Compared to native PE we found Ag-DLC-PE treated with high implantation energies (100 keV) to be even more susceptible for bacterial colonization. This finding is surprising, since DLC coatings of other materials than PE (e.g. steel, PVC) showed significant antibacterial potency in several investigations (Baba et al. 2013; Katsikogianni et al. 2006; Marciano et al. 2009). In consequence, low ion energies (10 keV) were used in the second testing group. The results showed clearly, that antibacterial potency increased with lower ion energies due to the deposition of Ag proximate to the surface and a therefore potentially higher concentration of released Ag^+^. Therefore an increased antimicrobial effect was determined not only on the surface but also in the surrounding medium. To identify which of the parameters (ion energy or fluence) might have major impact on Ag^+^ dissolution and consequently the antimicrobial effect of the coating the third testing group with rearranged sample features was conducted. We found a strong dependency of antibacterial activity and the fluence of Ag^+^ in the coatings. This led to the conclusion, that ion energy plays a minor role as long as low energies (e.g. 10 or 20 keV) are applied during Ag^+^ implantation.

Moreover, the conversion of the superficial PE by ion implantation might be beneficial with regard to mechanical properties compared to conventional surface coatings. Conversion of the superficial PE material to DLC-PE results in a gradient of conversed DLC, and thus reduces the risk of abrasive wear observed with certain DLC coatings on various metallic biomaterials.

This study involves several limitations. Ion concentrations in the surrounding medium were not measured. Thus, Ag^+^ release could not be quantified though antibacterial effectiveness of the surface modification was proven. A significant antibacterial effect of DLC-PE without integrated Ag, on the other hand could be ruled out in the present study (Table 1) and in our previous experiments (data not shown). Another limitation is that only two bacterial strains were used in this study. Although the investigated strains are of major importance in periprosthetic joint infections, antibacterial effect against other bacteria has to be investigated in future studies. In fact, several studies confirmed even higher bactericidal potency of Ag^+^ against Gram-negative compared to Gram-positive bacteria (Flores et al. 2013; Kim et al. 2007). Additionally, no influence of Ag-DLC on osseointegration was investigated. A negative effect on eukaryotic cells in this context could be of major interest in the clinical use of this antibacterial coating even though PE is not used in direct bone contact. However, this was not the scope of this proof of principle investigation. Further investigations are needed in order to clear whether the concentration and duration of delivery of the released Ag^+^ of Ag-DLC-PE is sufficient to avoid implant infection in vivo and how they interact with bony tissue.

Taken together, our findings strongly support further investigation of Ag-DLC conversion of PE for prophylaxis of implant-associated infections. Antibacterial effectiveness of Ag-DLC-PE has been demonstrated. The suitability of this surface modification for biomedical applications will be confirmed by wear tests and in vitro biocompatibility assessments.

## Abbreviations

PJI: periprosthetic joint infections
Ag: silver
Ag^+^: silver ion
DLC: diamond-like carbon
PE: polyethylene
DLC-PE: diamond-like carbon coating on polyethylene
Ag-DLC: silver incorporated diamond-like carbon coating
Ag-DLC-PE: silver incorporated diamond-like carbon coating on polyethylene
CFU: colony forming units
SD: standard deviation
